# *GsCHX19*.3, a member of cation/H^+^ exchanger superfamily from wild soybean contributes to high salinity and carbonate alkaline tolerance

**DOI:** 10.1038/s41598-017-09772-3

**Published:** 2017-08-25

**Authors:** Bowei Jia, Mingzhe Sun, Huizi DuanMu, Xiaodong Ding, Beidong Liu, Yanming Zhu, Xiaoli Sun

**Affiliations:** 1 Key Laboratory of Agricultural Biological Functional Genes, Northeast Agricultural University, Harbin, 150030 P.R. China; 2 Crop Stress Molecular Biology Laboratory, Heilongjiang Bayi Agricultural University, Daqing, 163319 P.R. China; 3 Department of Chemistry and Molecular Biology, University of Gothenburg, Box 462, Medicinaregatan, 9ES-413 90 Gothenburg, Sweden

## Abstract

Cation/H^+^ exchangers (CHX) are characterized to be involved in plant growth, development and stress responses. Although soybean genome sequencing has been completed, the CHX family hasn’t yet been systematically analyzed, especially in wild soybean. Here, through Hidden Markov Model search against *Glycine soja* proteome, 34 *GsCHXs* were identified and phylogenetically clustered into five groups. Members within each group showed high conservation in motif architecture. Interestingly, according to our previous RNA-seq data, only Group IVa members exhibited highly induced expression under carbonate alkaline stress. Among them, *GsCHX19.3* displayed the greatest up-regulation in response to carbonate alkaline stress, which was further confirmed by quantitative real-time PCR analysis. We also observed the ubiquitous expression of *GsCHX19.3* in different tissues and its localization on plasma membrane. Moreover, we found that *GsCHX19.3* expression in AXT4K, a yeast mutant lacking four ion transporters conferred resistance to low K^+^ at alkali pH, as well as carbonate stress. Consistently, in *Arabidopsis*, *GsCHX19.3* overexpression increased plant tolerance both to high salt and carbonate alkaline stresses. Furthermore, we also confirmed that *GsCHX19.3* transgenic lines showed lower Na^+^ concentration but higher K^+^/Na^+^ values under salt-alkaline stress. Taken together, our findings indicated that *GsCHX19.3* contributed to high salinity and carbonate alkaline tolerance.

## Introduction

Soil salt-alkalinity, one of the most important environmental stresses, not only reduces soil fertility, but also abates the amount of agricultural production. Salt-alkaline stress has severely limited the sustainable development of agriculture and economy. Also, with the growing population and increasing demand for food, soil salinization and alkalization has become one of the severest problems. Thus, it is necessary and urgent to study the molecular responses to salt-alkaline stress in crops.

As sessile organisms, plants have developed a variety of physiological and biochemical mechanisms to cope with saline-alkaline stress during their long-term evolution process. It has been well studied that under salt-alkaline stress, cation/proton antiporters (CPAs) serve to maintain lower cytoplasmic Na^+^ concentration and cellular pH homeostasis^1^. The CPA superfamily is structurally characterized by a conserved Na^+^/H^+^ exchanger domain, and could be divided into CPA1 and CPA2 subfamilies. The CPA1 subfamily consists of NHAP and NHX clades, while CPA2 is composed of NHA, KEA, and CHX clades^2^. Among them, CPA1 have been suggested to function in exchanging Na^+^ or Li^+^ for H^+^, and maintaining pH balance under adverse conditions, such as ABA, salt and hyperosmotic stresses^3, 4^. Compared with CPA1, research concerning the CPA2 family is very limited until now. Recently, studies of KEAs from the CPA2 family have been gradually begun. For instance, *AtKEAs* were functionally characterized as K^+^/H^+^ antiporters and functioned under high K^+^ stress^5^. However, studies about CHXs are limited in plant growth and development^6, 7^, while little is known about their function in response to salt and alkaline stress.

In *Arabidopsis*, the CHX family was divided into five groups (Group I-V)^8^. Group IV was the largest one, and consisted of 8 members (*AtCHX15–21* and *AtCHX23*). Among them, *AtCHX16–20* were all endomembrane transporters^9–11^. *AtCHX 16–19* shared overlapping roles in reproduction and seed development^11^. *AtCHX20* was highly expressed in guard cells, and functioned in osmoregulation of stomatal open by regulating K^+^ movement and pH homeostasis^12^. *AtCHX21* was a putative Na^+^ transporter, regulating Na^+^ balance in xylem and Na^+^ accumulation in leaves^13^. *AtCHX23* functioned in cytosol pH adjustment. In *atchx21chx23* mutants, pollen tubes failed to target ovules, which resulted in impaired pollen fertility^6^. However, date to now, studies on CHXs are mainly limited in *Arabidopsis*, the function of CHXs from other species, in carbonate stress responses is still unclear.

*Glycine soja* is a related species of *Glycine max*, which can survive in severely saline-alkaline areas^14^. In previous studies, from a total of 345 wild soybean lines, we screened out *G. soja* 07256, which showed the highest resistance to carbonate stress^14^. By using microarray and RNA-seq data, we constructed the gene expression profiles in response to carbonate alkaline stress^15^, and further excavated several stress resistant genes, such as *GsCBRLK* ^16^, *GsSKP21* ^17^, *GsACA1* ^18^, *GsGSTU13* ^19^ and so on. Considering the potential roles of *CHXs* in stress responses, in this study, we focused on the CHX family genes of *G.soja*. A total of 34 *GsCHXs* were identified and clustered into five groups. Expression profiles of Group IVa members were investigated in detail, and one of them *GsCHX19.3* was demonstrated to mediate K^+^ uptake and positively regulate plant responses to high salt and carbonate alkaline stresses.

## Results

### Genome-wide identification and characterization of the CHX family genes in *G. soja*

**Figure 1:**
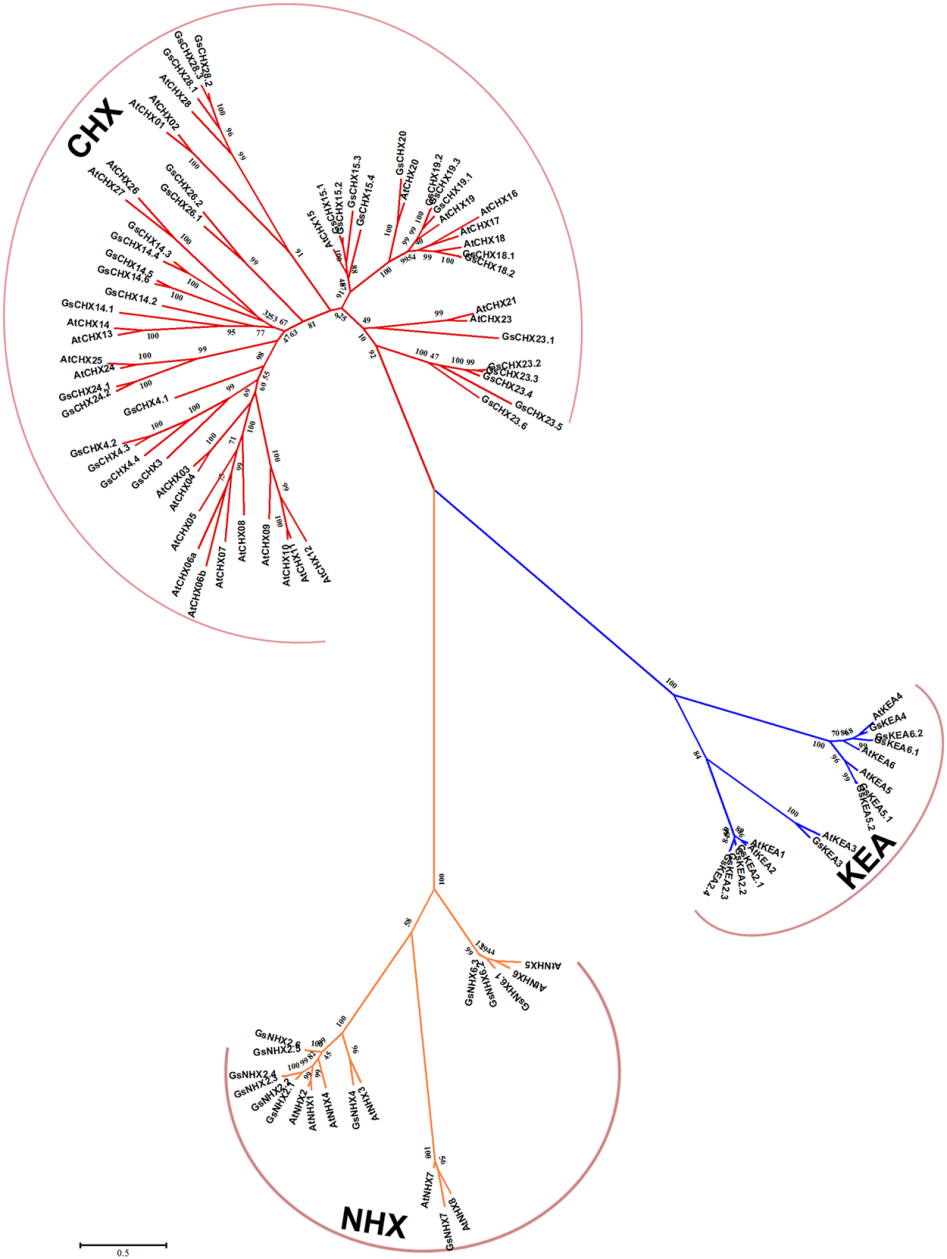
Phylogenetic tree of CPA family from *Glycine soja* and *Arabidopsis thaliana*. The evolutionary relationship of 97 CPA genes was determined by MEGA6.0 using neighbor-joining method with a bootstrap of 1000. The CPA family was subdivided into 3 subfamilies: CHX (red), KEA (blue), and NHX (yellow) clades. Accession numbers of protein sequences are listed in Supplementary Table 1.

To identify the CHX family genes in *G. soja*, we carried out Hidden Markov Model (HMM) search against *G. soja* proteome to identify all CPA superfamily members. A total of 55 non-redundant CPA genes were obtained, and were used to construct a phylogenetic tree with *Arabidopsis* CPAs (AtCHXs^8^, AtKEAs^5^ and AtNHXs). Phylogenetic analysis revealed that 34 of the *G. soja* CPA genes formed a cluster with AtCHXs, belonging to GsCHXs (Fig. 1). Then these *GsCHXs* were designated according to their homologous and evolutionary relationship to *AtCHXs*. Basic information of all *GsCHXs* (including gene name, DNA, CDS, protein length, molecular weight, isoelectric point, transmembrane number, predicted localization and Na^+^/H^+^ exchanger domain) is provided in Table S1. Notably, all these GsCHXs contained a Na^+^/H^+^ exchanger domain in their N-terminus. What’s more, they all shared 7–13 transmembrane domains and were predicted to localize on plasma membrane or other membrane-containing organelles, indicating their potential function as membrane transporters.

### Phylogenetic and structural analysis of soybean *CHXs*

**Figure 2:**
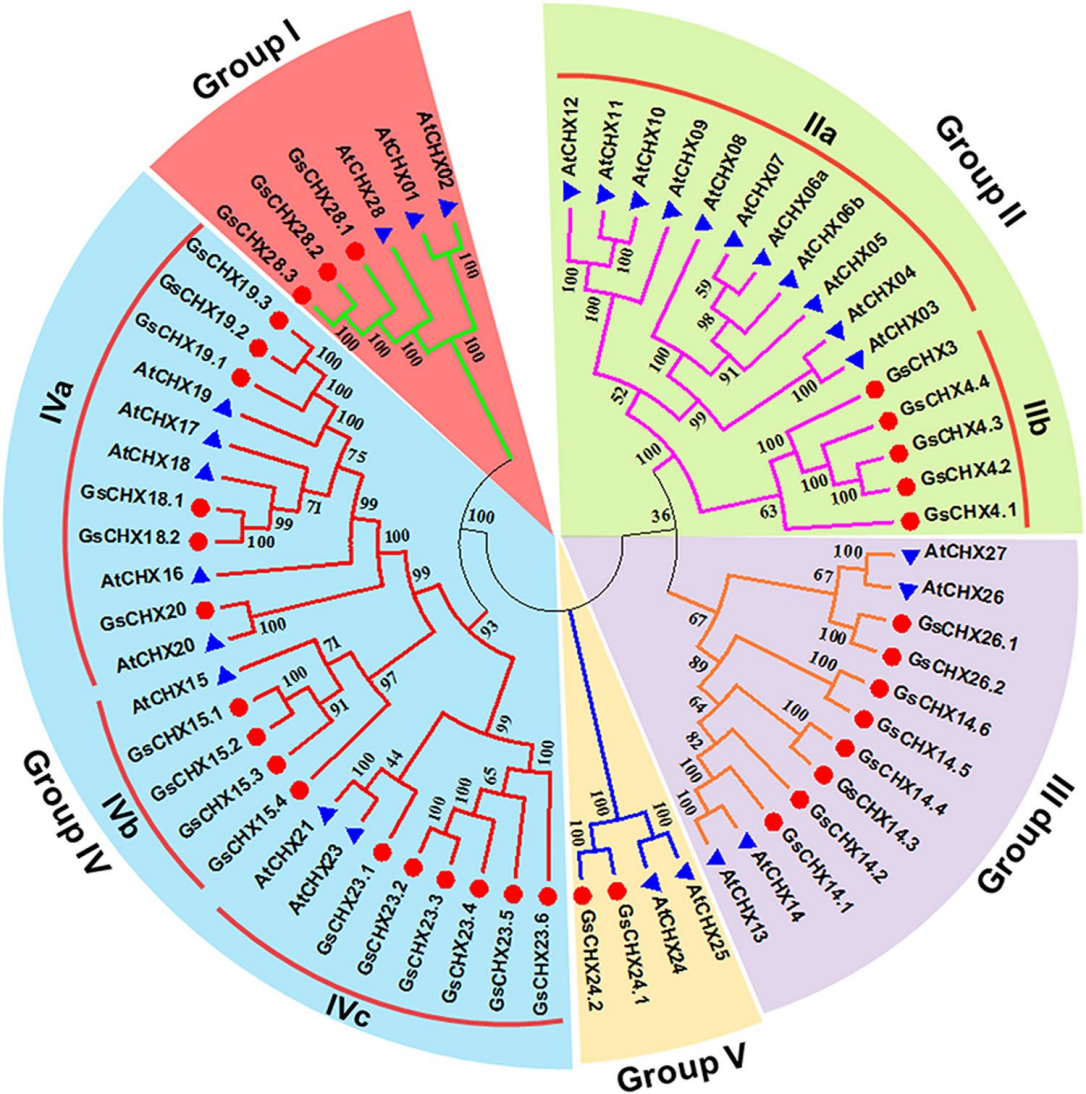
The phylogenetic tree of cation/H^+^ exchangers from *Glycine soja* and *Arabidopsis thaliana*. The phylogenetic tree was produced by MEGA 6.0 software based on the full-length amino acid sequence. The neighbor-joining method was used with 1000 bootstrap replications. CHXs were divided into five groups (Group I, II, III, IV and V) and separated by different colors. Genes from different species are marked with different bullet point colors.

To explore the evolutionary relationship of *GsCHXs*, a phylogenetic tree was constructed with CHX protein sequences from *G. soja* and *Arabidopsis*. As depicted in Figs 1 and 2, similar to *AtCHXs*, *GsCHXs* were also classified into five groups (Group I–V)^8^. Among them, Group II could be further divided into two subgroups Group IIa (AtCHX3–12) and Group IIb (GsCHX3–4.4). Group IIa were previously reported to be specific to *Arabidopsis* ^2^, we speculated that Group IIb might be peculiar to soybean, indicating the diversification of *CHXs* during soybean speciation.

**Figure 3:**
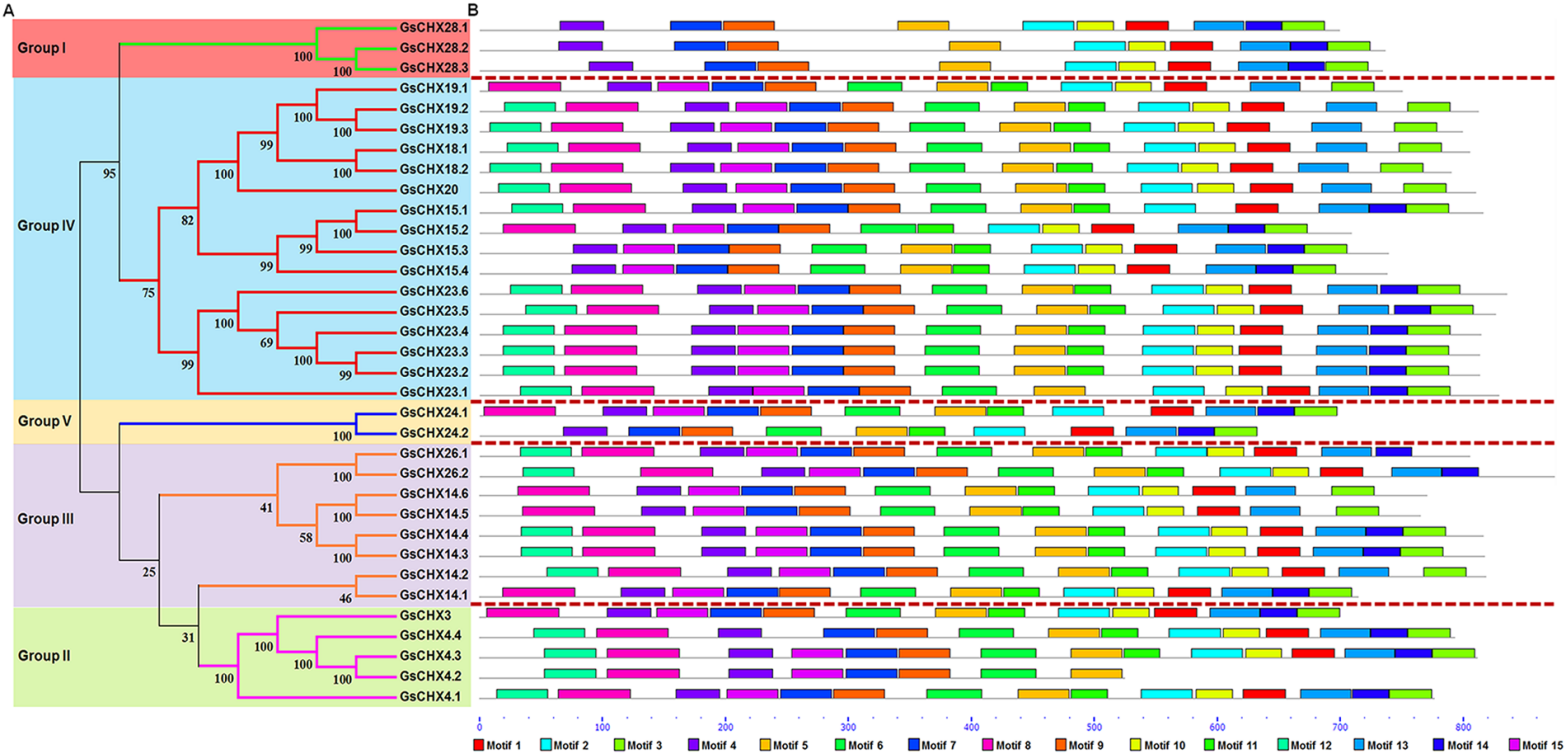
Phylogenetic relationship and putative representation of the conserved motifs of GsCHXs. (**A**) The unrooted tree was produced by MEGA 6.0 software using the full-length amino acid sequences of 34 GsCHXs by the neighbor-joining method with 1000 bootstrap replications. Genes from different groups are classified by different colors. (**B**) Schematic representation of the conserved motifs in GsCHXs. Each colored box represents a motif. The length of the protein a motif can be estimated using the scale at the bottom. A detailed motif introduction is shown in Supplementary Figure 1.

To further examine the conservation of GsCHX protein sequences and structures, the MEME program was used to predict conserved motifs (Figs 3 and S1). Expectedly, GsCHXs displayed high similarity in protein sequences, and GsCHXs within each individual group shared common motif architecture. Among the checked 15 motifs, motif 4, 6, 7, 8, 9, 12 and 15 in the N-terminus were fragments of the well-known Na^+^/H^+^ exchanger domain. Motif 2, 5, 10 and 11 composed the AANH-like domain^7^, which is beside the C-terminus of Na^+^/H^+^ exchanger domain. Besides, another four conserved motifs (Motif 1, 3, 13, 14) were identified in the C terminus, but their functions were still unknown. Taken together, CHX family was evolutionarily conserved in terms of motif distribution, especially in each individual group.

### Expression profiles of GsCHXs under carbonate alkaline stress

**Figure 4:**
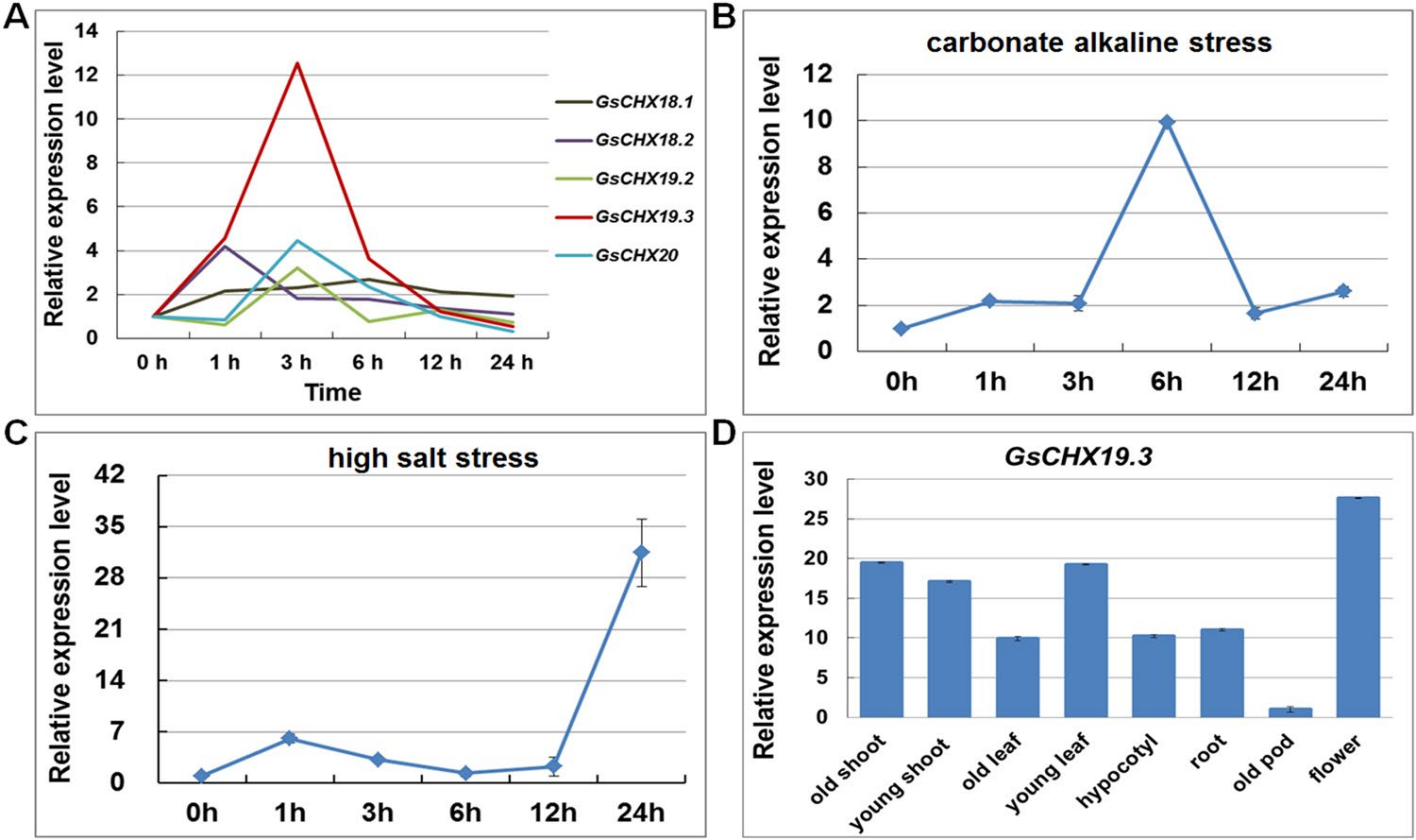
Expression analysis of *GsCHX19.3*. (**A**) Relative expression levels of five *GsCHXs* from Group IVa based on our previous RNA-seq data. (**B**,**C**) qRT-PCR results showing expression levels of *GsCHX19.3* under carbonate alkaline and high salt stress in *G. soja* roots. (**D**) Expression levels of *GsCHX19.3* in different tissues and organs in *G. soja*. Relative transcript levels were determined by qRT-PCR analysis with *GsGAPDH* (accession: KN656371.1) as an internal control. The mean values from three fully independent biological repeats and three technical repeats are shown.

The CHX family genes are found to be involved in plant growth and development and it has been documented that CHX family genes functioned in response to adversity stress^4, 5, 12, 16^. However, little is known about the roles of GsCHXs in carbonate alkaline stress. Hence, we analyzed the expression profiles of GsCHX family genes under carbonate alkaline stress (50 mM NaHCO_3_, pH8.5) based on our previous RNA-seq data (Fig. S2 and Table S2). As depicted in Figs S2 and 4A, only five genes from Group IVa (*GsCHX18.1, GsCHX18.2, GsCHX19.2, GsCHX19.3, GsCHX20*) exhibited high expression levels and responded to carbonate alkaline stress (Figs S2 and 4A). Among them, *GsCHX19.3* exhibited the greatest induction by carbonate alkaline stress, and the value was up to 12 fold (Fig. 4A). Therefore, we focused on *GsCHX19.3* for further study.

### Examination of *GsCHX19.3* expression characteristics in *G. soja* seedlings

To get better understanding of *GsCHX19.3* expression, we firstly confirmed the induction of *GsCHX19.3* under carbonate alkaline stress. In accordance with the RNA-seq data, *GsCHX19.3* expression was greatly induced by carbonate alkaline stress (50 mM NaHCO_3_) (Fig. 4B). After carbonate alkaline treatment, the transcript levels of *GsCHX19.3* increased within 6 h to nearly 10 folds, and then decreased at 12 h (Fig. 4B). Considering the fact that high salt stress always occurred simultaneously with carbonate alkaline stress, we further checked the expression of *GsCHX19.3* under high salt treatment (200 mM NaCl). As expected, the expression of *GsCHX19.3* showed an obvious increase after high salt treatment (Fig. 4C). Overall, results represented here showed that *GsCHX19.3* expression was greatly induced by both carbonate alkaline and high salt stresses (Fig. 4B,C), suggesting an important role of *GsCHX19.3* in plant responses to salt-alkaline stress.

It has been reported that CHXs exhibited diverse expression patterns in different tissues during plant growth and developmental processes^8, 13^. Then we carried out qRT-PCR to detect the expression patterns of *GsCHX19.3* in different tissues of *G. soja* (including old shoot, young shoot, old leaf, young leaf, hypocotyl, root, old pod and flower). The results showed that *GsCHX19.3* expressed in all detected tissues, and exhibited higher expression levels in flower and young leaf (Fig. 4D). This result was accordant with that of *AtCHX19*, which also displayed high expression levels in flower and leaf^8^.

### GsCHX19.3 localized to the plasma membrane in onion epidermal cells

**Figure 5:**
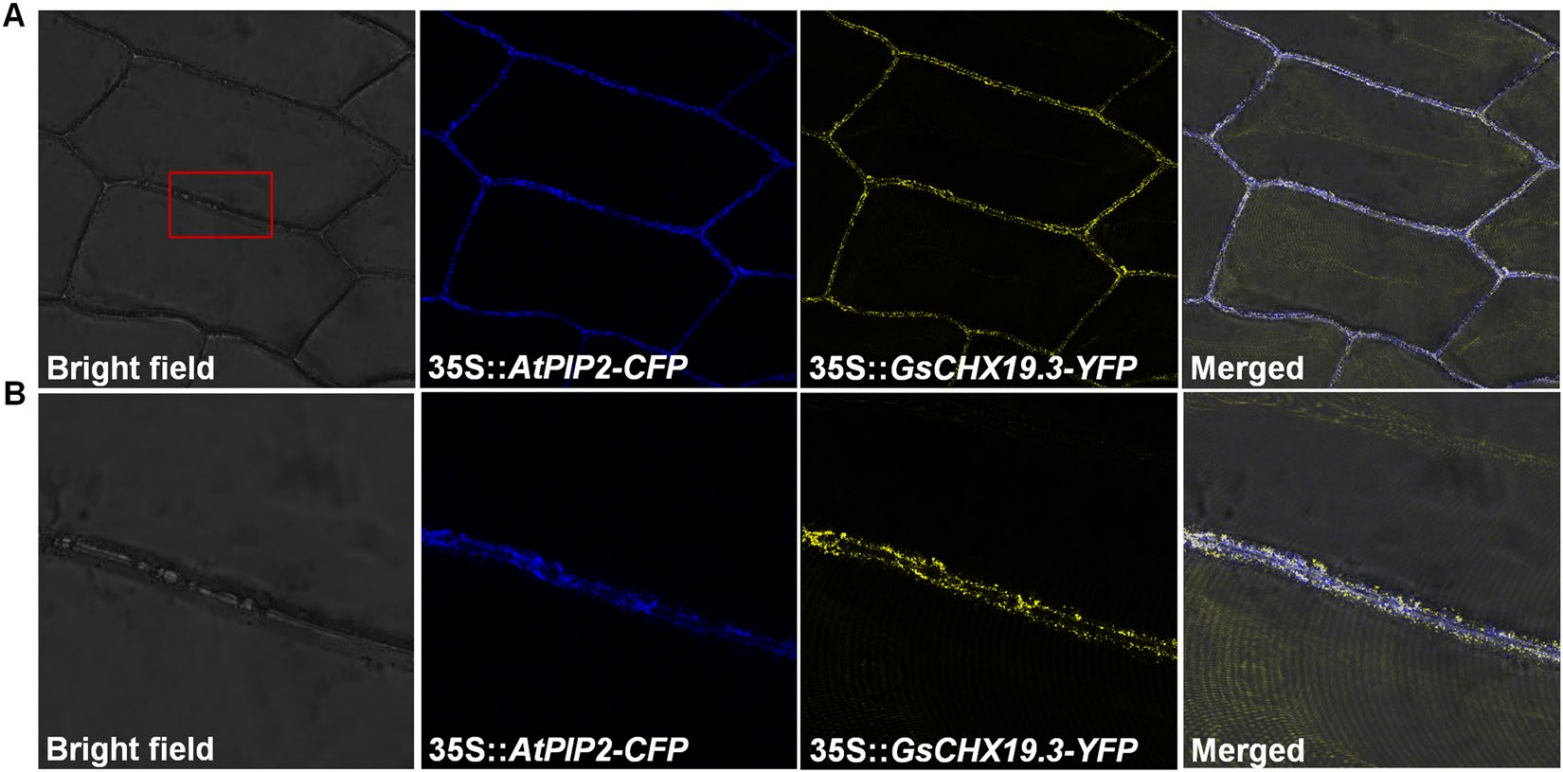
Subcellular localization of GsCHX19.3. (**A**) GsCHX19.3-YFP co-localized with AtPIP2-CFP at plasma membrane in the onion epidermal cells. (**B**) An enlarged section of onion plasma membrane co-expressing GsCHX19.3-YFP and AtPIP2-CFP. The GFP and CFP signal were checked by using a confocal laser-scanning microscope. The panel showed bright-field illumination of the onion epidermal cells and confocal images of the YFP signal (yellow), the CFP signal (blue) and the GFP–CFP merged signal.

As ion transporters, CHXs usually localize on the endomembrane system to regulate cation and pH homeostasis. Hence, to localize GsCHX19.3 protein, we constructed expression vector with *GsCHX19.3* tagged at the N-terminus of yellow fluorescent protein (YFP). The AtPIP2-CFP construct was used as plasma membrane marker^20^. Then GsCHX19.3-YFP and AtPIP2-CFP were transiently co-expressed in onion epidermal cells. As shown in Fig. 5, the signal of GsCHX19.3-YFP protein was exactly overlapped with AtPIP2-CFP on plasma membrane, which was consistent with the result of PSORT prediction (Table S1). This finding was also consistent with previous report that AtCHX19 protein localized on plasma membrane^21^.

### Functional complementation assays of *GsCHX19.3* in yeast mutant

**Figure 6:**
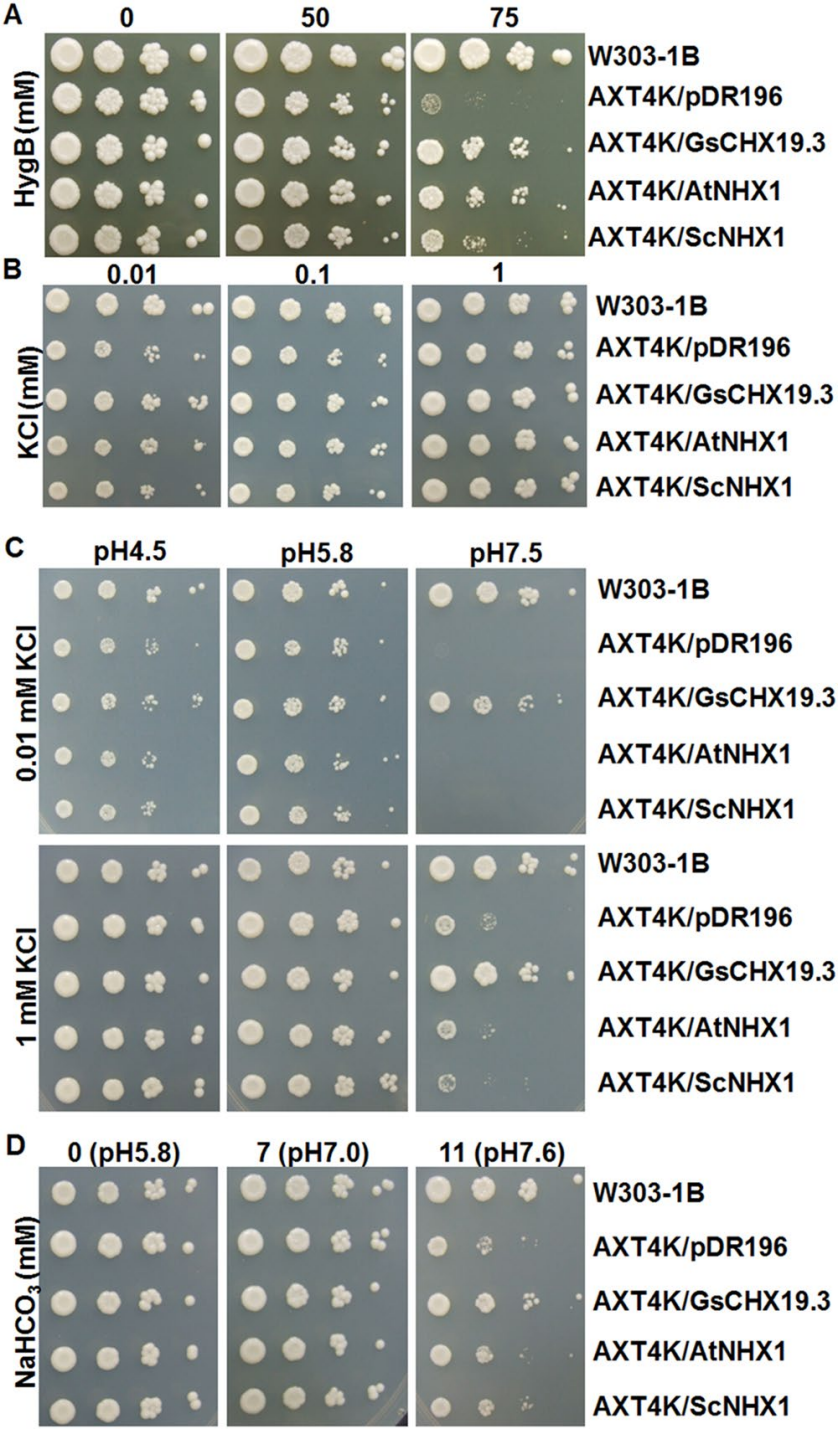
Functional complementation of *GsCHX19.3* in yeast mutant AXT4K. Culture concentration of transformed yeasts was normalized in water to OD_600_ = 0.4. 10-fold serial dilutions were spotted onto YPD plates with different concentrations of hygromycine B (**A**), or AP plates containing different concentrations of KCl (**B**), or AP plates with 0.01 mM or 1 mM KCl at different pH values (**C**), or AP plates with different concentrations of NaHCO_3_ (**D**). Plates were placed at 30 °C, and pictures were taken after 3 days to show yeast growth performance.

To test the transport activity of *GsCHX19.3*, we performed the functional complementation analyses by using the *Saccharomyces cerevisiae* strain AXT4K. Strain AXT4K was generated by deleting plasma membrane Na^+^-ATPase (*ScENA1–4*), plasma membrane Na^+^, K^+^/H^+^ antiporter (*ScNHA1*), vacuolar Na^+^, K^+^/H^+^ antiporter (*ScNHX1*)^22^, and endoplasmic reticulum K^+^/H^+^ exchanger (*ScKHA1*)^10^ in the W303-1B background. AXT4K was sensitive to high salt and low potassium with alkali pH^5^. Besides, AXT4K was also sensitive to hygromycin B, an aminoglycosidic antibiotic produced by *Streptomyces hygroscopicus*, which inhibits protein synthesis in both prokaryotic and eukaryotic cells^23^. It has been reported that *ScNHX1* and *AtNHX1* expression could restore AXT4K tolerance to hygromycin B^5^. In this study, to express *GsCHX19.3* in AXT4K, its CDS region was cloned into the pDR196 expression vector and introduced into AXT4K. Growth performance of yeast cells revealed that *GsCHX19.3* expression in AXT4K could also recover its hygromycin B resistance (Fig. 6A), indicating GsCHX19.3 could function normally in AXT4K.

We further checked the effects of *GsCHX19.3* on ion transporting. To do this, transformed yeasts were grown on Arg phosphate (AP) or YPD medium with different levels of KCl and NaCl. As shown in Fig. S3, *GsCHX19.3* expression didn’t restore yeast tolerance to high K^+^ and Na^+^ stress under normal pH condition. We also observed that all transformants showed no growth difference on mediums with 0.01 (low K^+^), 0.1 (low K^+^) and 1mM KCl (normal) at normal pH (pH5.8) (Fig. 6B). These findings suggested *GsCHX19.3* didn’t function under either low K^+^, or high K^+^, or high Na^+^ stress.

Previous studies have shown that *AtCHX19* functioned at low K^+^ alkalinity condition^9, 11^. Hence, to further explore the function of *GsCHX19.3* at low K^+^ and high pH, the transformed yeasts were grown on low-KCl AP medium (0.01 and 0.1 mM KCl) at either pH4.5 (acidic), or pH5.8 (normal), or pH7.5 (alkali). As shown in Fig. 6C, all yeasts exhibited no growth difference on normal AP medium (pH 5.8 with 1 mM K^+^). When pH lowered to 4.5, no significant difference was observed for the growth performance of different transformed yeasts. However, when the pH value was up to 7.5, only *GsCHX19.3* expressed yeasts grew as well as W303-1B. Especially, only *GsCHX19.3* and W303-1B could survive on AP medium with 0.01 mM KCl at pH7.5. Based on these results, we believe that *GsCHX19.3* functions as an active K^+^ uptake transporter under alkali pH.

In the view of induced expression of *GsCHX19.3* under carbonate stress, we further tested the yeast growth on YPD medium with 0, 7 or 11 mM NaHCO_3_ (Fig. 6D). When supplemented with 11 mM NaHCO_3_, W303-1B and *GsCHX19.3* transformed yeasts grew much better than other yeast cells, which implied that *GsCHX19.3* expression in AXT4K could also facilitate yeast tolerance to carbonate stress. In conclusion, results from yeast complementation assays revealed that *GsCHX19.3* functioned as a K^+^ uptake transporter at alkali pH, and also participated in carbonate stress responses in yeast cells.

### *GsCHX19.3* overexpression in *Arabidopsis* increased plant tolerance to salt-alkaline stress

**Figure 7:**
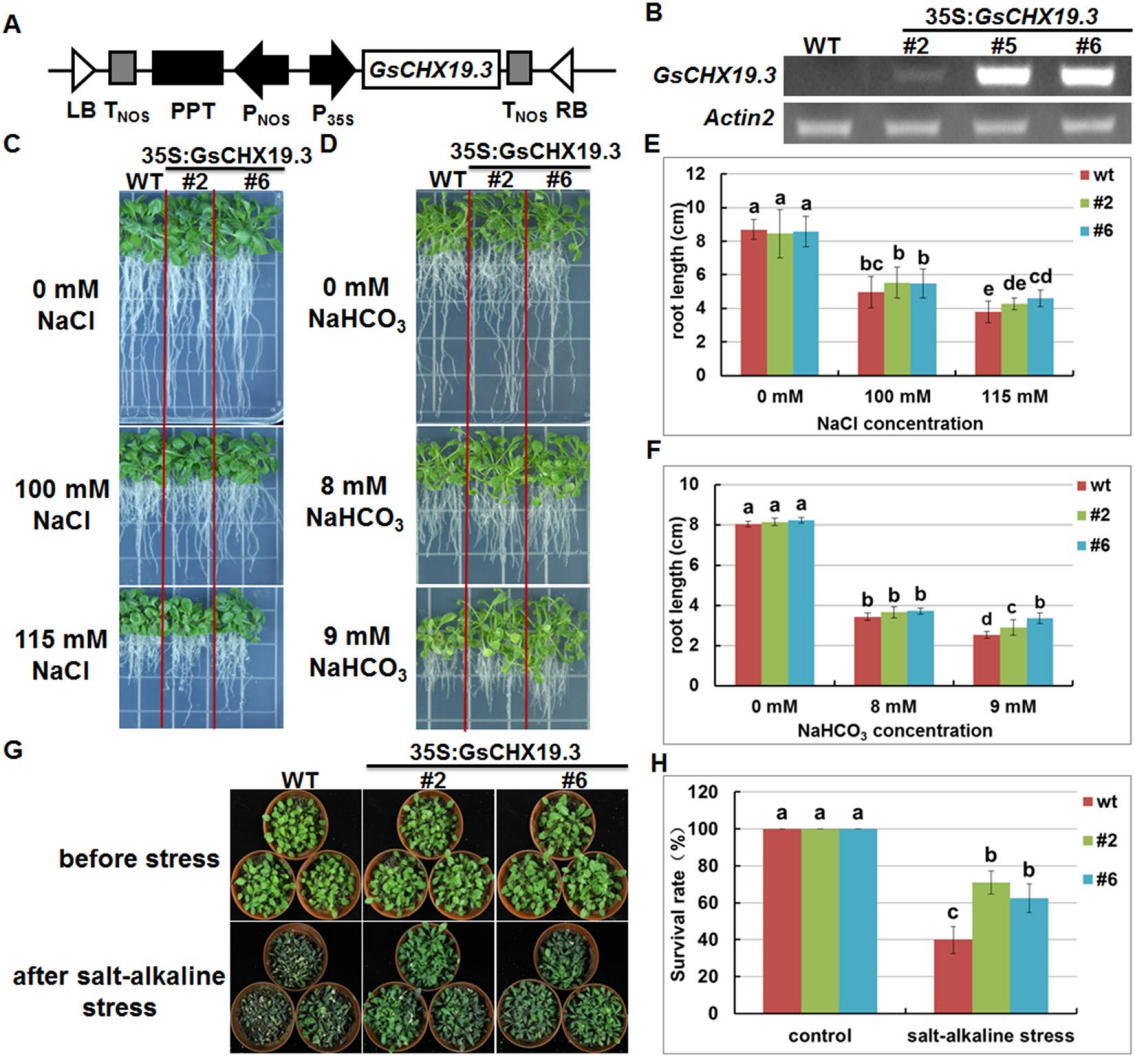
GsCHX19.3 positively regulates saline-alkaline stress tolerance in plants. (**A**) Construction of *GsCHX19.3* overexpression vector. (**B**) Characterization of *GsCHX19.3* overexpression transgenic lines. WT was used as a control and Actin 2 was used as an internal standard. (**C**,**D**) Phenotypes of WT and OX seedlings under high salt (**C**), or carbonate alkaline stress (**D**). (**E**,**F**) Primary roots of WT and OX seedlings under high salt (**E**), or carbonate alkaline stress (**F**). The 11-day-old seedlings grown on normal 1/2MS medium were transferred to new plates supplemented with 0, 100, 115 mM NaCl or 0, 8, 9 mM NaHCO_3_. Photographs were taken after 7 days. Fifteen seedlings of each line were used for each experiment. (**G**) Phenotypes of WT and OX adult seedlings under carbonate alkaline. (**H**) Survival rates of WT and OX adult seedlings under carbonate alkaline. The 4-week-old soil-grown seedlings were watered with 150 mM NaHCO_3_ (pH 9.0) solution. Photographs were taken after 15 days. Data are means (±SE) of three replicates (P < 0.05, one-way ANOVA).

The stress induction of *GsCHX19.3* expression in *G. soja* and its functional complementation in yeast mutant AXT4K preliminarily indicated its potential roles in K^+^ uptake and stress responses. Hence, to further evaluate the function of *GsCHX19.3* in plants, its CDS region was cloned into pCAMBIA330035Su^24^, and transformed into wild type *Arabidopsis* (Columbia background) through floral dip method (Fig. 7A). Three overexpression (OX) transgenic lines were identified through semi-quantitative RT-PCR analyses (Fig. 7B), and two of them (#2 and #6) with different expression levels were used for further phenotypic assays.

By using the root length assay, we checked whether *GsCHX19.3* overexpression in WT could alter high salt (NaCl) and carbonate alkaline (NaHCO_3_) tolerance. As shown in Fig. 7C,D, all seedlings (WT, and OX lines) showed similar growth under normal condition. Under salt and alkaline stresses, primary roots of OX seedlings were significantly longer than those of WT (Fig. 7E,F) (p < 0.05 by one-way ANVOA).

To further evaluate the salt-alkaline tolerance at the adult stage, the 4-week-old WT and OX plants were watered with 150 mM NaHCO_3_ (pH = 9.0). After salt-alkaline treatment for 15 days, WT exhibited chlorosis and wilting, even death, but OX plants appeared relatively healthy (Fig. 7G). Quantification analysis of the survival rate revealed that only 39.8% of WT plants survived, while the survival rates for OX lines were 70.9% (line 2) and 62.5% (line 6) (Fig. 7H) (p < 0.05 by one-way ANVOA). Above all, *GsCHX19.3* plays a positive role in response to salt-alkaline stress in plants.

### *GsCHX19.3* mediates K^+^ uptake and Na^+^ excretion under carbonate alkaline stress in plants

**Figure 8:**
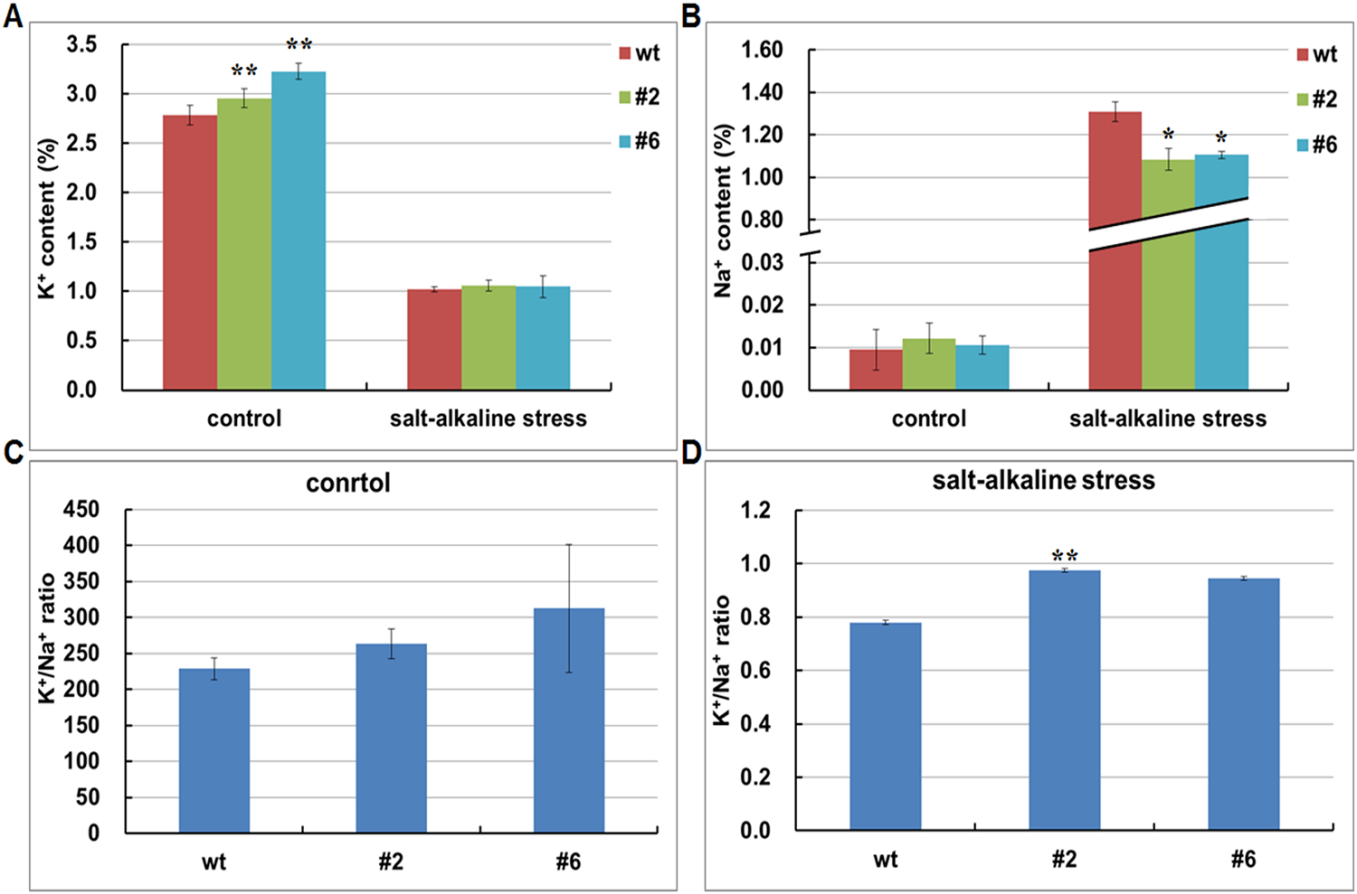
*GsCHX19.3* mediates K^**+**^ uptake and Na^**+**^ excretion under carbonate alkaline stress in plants. K^+^ content (**A**) and Na^+^ content (**B**) in leaves of WT and OX under normal condition and carbonate alkaline stress. K^+^/Na^+^ ratio of WT and OX under normal condition (**C**) and carbonate alkaline stress (**D**). Data are means (±SE) of three replicates. *P < 0.05; **P < 0.01 by Student’s t-test.

During the long term evolution, plants have developed unique mechanisms to resist salt-alkaline stress. When suffering salt stress, plants would promote K^+^ absorbtion in order to alleviate Na^+^ toxic and maintain K^+^/Na^+^^25, 26^. Considering the function of CHXs in cation transporting, we further examined the Na^+^ and K^+^ content in WT and OX plants under both normal and salt-alkaline stress. Under normal growth condition, *GsCHX19.3* OX lines exhibited significantly higher K^+^ content than WT, but showed similar Na^+^ accumulation to WT (Fig. 8A,B) (*p < 0.05, **p < 0.01 by Student’s *t-test*). This implied a positive role of *GsCHX19.3* in K^+^ uptake in plants.

After carbonate alkaline treatment, all plants showed an obvious decrease in K^+^ content, but a great increase in Na^+^ accumulation (Fig. 8A,B) (*p < 0.05, **p < 0.01 by Student’s *t-test*). Compared with WT, OX lines displayed much lower Na^+^ content under stress treatment (Fig. 8B) (*p < 0.05, **p < 0.01 by Student’s *t-test*). However, the K^+^ content of WT is similar to that of OX lines (Fig. 8A). As a consequence, under normal condition, WT displayed lower K^+^/Na^+^ ratio, compared with OX lines (Fig. 8C). After carbonate alkaline treatment, all plants showed decreased K^+^/Na^+^ ratio, but the K^+^/Na^+^ ratio of OX was remarkably higher than WT (Fig. 8D) (**p < 0.01 by Student’s *t-test*).

In conclusion, above results indicated that *GsCHX19.3* overexpression could increase K^+^ absorbtion under normal condition, and reduce Na^+^ absorbtion or increase Na^+^ excretion to alleviate ion poison caused by salt-alkaline stress.

## Discussion

Till now, soil salinization-alkalization has become a worldwide problem limiting agricultural production. Salt stress results in increased cytoplasmic Na^+^ concentration and disrupts ion homeostasis. Consequently, excess Na^+^ inhibits enzyme activity^27^. Therefore, once upon salt stress, plants have to start a series of signaling pathways to maintain low Na^+^ but high K^+^ content^26, 28–30^. For this reason, research on CHX family is of great importance to understand the responsive mechanism of saline-alkaline stress.

The CHX family has been gradually studied in *Arabidopsis* ^2^, rice^8, 31^, and *Physcomitrella patens* ^32^. However, no systematical study was performed for CHXs in legume plants. *Glycine soja*, as a sibling species of *Glycine max*, exhibits high carbonate tolerance and has become one of the most popular materials to study saline-alkaline stress signaling pathway^21, 33, 34^. In this study, we identified a total of 34 *CHXs* in soybean genome and found that soybean possessed about 1.2 times *CHXs* as many as *Arabidopsis*. Gene duplications were one of the reasons to force gene family expansion and generate homologous genes with similar structures and functions^35^. Soybean experienced twice gene duplications during evolution, but only once in *Arabidopsis* ^36^, which probably contributed to more *CHXs* in soybean than *Arabidopsis*.

Studies have shown that CHX family contains a Na^+^/H^+^ exchange domain in the N terminus and an AANH_like domain close to the C terminus^7, 8, 12^. Besides, in this study, through searching conserved motifs, we also found another conserved domain in the long C tail of soybean CHXs (Fig. 3B). Although the function of this domain was mysterious, we posited that it might be involved in phosphorylation or protein localization, like NHX family^37^. Previous reports also showed that deletion of *AtCHX17* C terminus inhibited its localization in endomembrane system^11^. Here, we proved that *GsCHX19.3* also localized on plasma membrane in plants (Fig. 5). Therefore, further experiments are needed to investigate the role of the C-terminal domain of *GsCHX19.3* in protein localization or activity.

Date to now, studies on CHX family genes were mainly focused on plant growth and development, but it has been reported that CHX family genes responded to adversity stress^6, 11, 12, 32, 34, 38–40^. Combined with our previous RNA-seq data^15^, we found that expression of five *GsCHXs* from Group IV responded to carbonate alkaline stress (Fig. S2). Interestingly, even though three *GsCHX19s* showed high sequence identity and close evolutionary relationship, only *GsCHX19.3* displayed a greatly increased expression under carbonate alkaline treatment (Fig. 4A). Further qRT-PCR results verified its up-regulated expression under both carbonate alkaline and high salt stress (Fig. 4B,C). Besides, *AtCHX16/17/18/19* from Group IV in *Arabidopsis* were all highly expressed in flower, leaf and root^8^. Here, we also found that *GsCHX19.3* was highly expressed in flower and leaf (Fig. 4D), indicating its roles in reproductive tissue development. Above findings suggested the involvement of *GsCHX19.3* in plant responses to salt-alkaline stress.

In this study, we further demonstrated the positive role of *GsCHX19.3* in salt-alkaline stress responses by mediating ion transport. To identify the ion transport activity and specificity, we used a yeast mutant AXT4K, in which the plasma membrane Na^+^-ATPase (*ScENA1-4*), plasma membrane Na^+^, K^+^/H^+^ antiporter (*ScNHA1*), vacuolar Na^+^, K^+^/H^+^ antiporter (*ScNHX1*)^22^, and endoplasmic reticulum K^+^/H^+^ exchanger (*ScKHA1*)^10^ were knockout. Here, we proved that *GsCHX19.3* expression could rescue the growth of AXT4K on the medium with normal K^+^ (1 mM) or low K^+^ (0.01 mM) at pH 7.5 (Fig. 6C). In previous studies, CHXs were functionally associated with K^+^ transport, especially when K^+^ deficiency met with high external pH^8, 11, 12, 32^. We also showed that *GsCHX19.3* overexpression in *Arabidopsis* increased the K^+^ uptake under normal growth condition (Fig. 8A). When K^+^ was limited, active transport through H^+^-cotransporters is needed to assist K^+^ uptake^41^. Thus, when the medium pH was slightly alkaline, cells could use a K^+^/H^+^ antiporter to accumulate K^+^ in cytosol and release H^+^ to extracellular to remit the alkaline damage.

Interestingly, *GsCHX19.3* expression in AXT4K couldn’t confer tolerance to high KCl or NaCl, even it made AXT4K more sensitive to high salt (Fig. S3). Similarly, previous studies have shown that *AtCHX20* and *AtCHX17* expression in yeast mutants also led to increased sensitivity to high salt stress^5, 12^. In contrast, NHX1 from grape was able to rescue the growth of *nhx1* yeast mutant under high salt stress^42^, and AtKEAs could mediate K^+^ transport under high K^+^ at normal pH (pH 5.8)^5^. Thus, the function of *GsCHX19.3* was distinct from that of NHX and KEA.

However, in plants, high salt stress up-regulated the expression of *GsCHX19.3* (Fig. 4C), and its overexpression also enhanced salt tolerance of transgenic *Arabidopsis* (Fig. 7C,E). Hence, it was worth noting that *GsCHX19.3* functioned under salt stress in plants, which was different from yeast. Previous studies have shown that H^+^ pump could generate pH gradient and thereby drive secondary transporters^43, 44^. Recent studies showed that the proton pumped out by H^+^-ATPase could act as substrates of SOS1 (an NHX subfamily member) to promote Na^+^ efflux, and this cooperation thereby is critical for salt stress responses at high pH^45^. Thus, we speculated the phenomenon of decreased Na^+^ content found in transgenic *Arabidopsis* might be secondary effects of *GsCHX19.3* transporter activity. Therefore, further work need be carried out to identify whether *GsCHX19.3* could cooperate with other genes to defense salt stress in plants.

We further suggested the biological function of *GsCHX19.3* under carbonate alkaline stress. First, AXT4K expressed *GsCHX19.3* grew much better than AXT4K under NaHCO_3_ stress. At the same time, *GsCHX19.3* overexpression in *Arabidopsis* also enhanced carbonate tolerance (Fig. 7D,F,G,H). As we know, Na^+^ concentration in cytoplasm of plant cells will dramatically increase under stress condition^46^. On one hand, plants will take effective measures to exclude excess cytoplasmic Na^+^ and lower cytoplasmic Na^+^ concentration. On the other hand, plants absorb more K^+^ to maintain the K^+^/Na^+^ balance. In this study, we found that carbonate alkaline stress increased cytoplasmic Na^+^ content, but *GsCHX19.3* OX lines showed lower Na^+^ content than WT (Fig. 8B). This finding suggested that *GsCHX19.3* overexpression could help to exclude cytoplasmic Na^+^, and result in a relative higher K^+^/Na^+^ values under salt-alkaline stress (Fig. 8D). However, till now, no electrophysiology evidence for K^+^, Na^+^ transporting by CHX proteins was reported. Further studies are needed to directly characterize the ion transport activity and specificity of GsCHX19.3 by using electrophysiology technologies, such as two-electrode, giant-patch, patch-clamp and double-barreled proton-selective microelectrode.

## Materials and Methods

### Identification and bioinformatics analysis of *G. soja* CHX gene family

The whole genome and proteome sequence of *G. soja* were obtained from NCBI Genome (https://www.ncbi.nlm.nih.gov/genome/13239)^34^. To identify *GsCHXs*, protein sequences of *Arabidopsis* CPA family (28 AtCHXs^2^, 6 AtKEAs^5^ and 8 AtNHXs) were used to build a Hidden Markov Model (HMM) profile by using HMMER 3.0. Then the HMM profile was used in local searches of *G. soja* proteome. All obtained CPA protein sequences from *G. soja* were compared with *Arabidopsis* CPA protein sequences by alignment with Clustal X and analyzed with MEGA6.0^2, 47^.

These proteins were examined for the existence of an intact Na^+^/H^+^ exchanger domain (Pfam: PF00999) by Pfam (http://pfam.xfam.org/), and were named according to their similarity to *Arabidopsis* homologous genes and phylogenetic relationship. SOSUI (http://harrier.nagahama-i-bio.ac.jp/sosui/sosui_submit.html) and PSORT (http://psort.hgc.jp/) were used to predict transmembrane domains and protein localization, respectively.

The phylogenetic tree with the neighbor-joining method with 1000 bootstrap values was constructed by MEGA 6.0. Expression values of GsCHX genes under 50 mM NaHCO_3_ treatment were acquired from our previous RNA-seq data^15^. The heatmap was built by using excel and adobe illustrator. The conserved motifs of GsCHXs were analyzed by MEME (http://meme.nbcr.net/meme/cgi-bin/meme.cgi).

### Quantitative Real-Time PCR

*G. soja* (07256) was grown and treated as described^14^. 50 mM NaHCO_3_ (pH = 8.5) for carbonate alkaline treatment, and 200 mM NaCl was used for high salt treatment.

RNAprep Pure Plant Kit (Tiangen, China) was used to extract total RNA and cDNA was generated using a SuperScript^TM^ III Reverse Transcriptase kit (Invitrogen, Carlsbad, CA, USA). Quantitative real-time PCR (qRT-PCR) was performed using Power SYBR Green PCR Master Mix (Applied Biosystems, Woolston, UK) on an ABI 7500 sequence detection system (Applied Biosystems). The gene specific primers were used for *GsCHX19.3* (5′-ACCCCTCAGACAACCCCG-3′ and 5′-TACGACGAATCGCACGCAT-3′) and *GsGAPDH* (accession: KN656371.1) (5′-GACTGGTATGGCATTCCGTGT-3′ and 5′-GCCCTCTGATTCCTCCTTGA-3′). Expression levels were calculated and normalized as described^48^.

### Subcellular localization of GsCHX19.3-YFP fusion protein in onion epidermal cells

*GsCHX19.3* without the stop codon was amplified with the forward primer (GGCTTAAUATGATGGCGACGAGTAACAAC) and the reverse primer (GGTTTAAUCCACTCGTTGGTGTGTCTGG), and then inserted into pCAMBIA2300YFPu vector to generate *GsCHX19.3*-YFP with the help of USER enzyme^24^. The plasma membrane marker (AtPIP2-CFP) was obtained from the Nebenführ Lab (http://nebenfuehrlab.utk.edu/markers/default.htm) ^20^. And these two vectors were introduced into *Agrobacterium tumefaciens* strain GV3101 together. Then the transformants were used to infect onion inner epidermal cells as described^49^. Confocal laser-scanning microscope Leica SP8 (Leica, Wetzlar, Germany) was used to detect the fluorescence at 514 nm (YFP) and 433 nm (CFP).

### Heterologous expression of *GsCHX19.3* in yeast mutant

*Saccharomyces cerevisiae* strains W303-1B (MATα *leu2-13 112*, *ura3-1*, *trp1-1*, *his3-11*, *ade2-1*, *can1–100*) and AXT4K (*ena1-4 Δ::HIS3*, *nha1 Δ::LEU2*, *nhx1 Δ::TRP1*, *kha1 Δ::KanMX6*) derived from W303-1B were kindly provided by Professor Quansheng Qiu^5, 50^. Untransformed strains were grown in YPDA medium (1% yeast extract, 2% peptone, 2% glucose and 0.003% ADE). Plasmids pDR196, pDR196-ScNHX1, and pDR196-AtNHX1 were also kindly obtained from Professor Quansheng Qiu^5^ as controls.

For functional complementation analysis in yeast, *GsCHX19.3* was cloned into pDR196 by using gene specific primers containing *Sal I* and *Xho I* sites (F: 5′-ACGCGTCGACATGATGGCGAC-3′, R: 5′-CCGCTCGAGTTAACTCGTTGGTGTG-3′). Plasmids were transformed into yeast cells by using lithium acetate method. Transformants were grown on selective AP medium as described^5^. For stress tolerance tests, concentration of yeast culture were normalized to OD_600_ =0.4 and then serially diluted. 1 μl aliquots of each 10-fold serial dilution were spotted onto AP plates supplemented with different concentration of KCl, or YPD plates supplemented with different contents of NaCl or hygromycine B, and incubated at 30 °C for 3 days. Drop test media should add 20 mM MES, and pH was adjusted with arginine^9^ or phosphoric acid^51^.

### Generation of *GsCHX19.3* overexpression lines

*Arabidopsis thaliana* Col-0 was used as wild type. *GsCHX19.3* was inserted into pCAMBIA330035Su vector^24^ using the following primers (F: 5′-GGCTTAAUATGATGGCGACGAGTAAC-3′, R: 5′-GGTTTAAUTTAACTCGTTGGTGTGTCTG-3′). Then WT *Arabidopsis* was transformed through floral dip method^52^, and T_1_ seeds were screened by 25 mg L^−1^ glufosinate ammonium (Sigma-Aldrich). The T_2_ seeds were harvested from individual plants and grown again, till homozygous lines were obtained. Transcript levels of *GsCHX19.3* in transgenic *Arabidopsis* were analyzed by semi-qRT-PCR using gene specific primers (F: 5′-ACCCCTCAGACAACCCCG-3′, R: 5′-TACGACGAATCGCACGCAT-3′). Actin 2 (AT3G18780) (F: 5′-TTACCCGATGGGCAAGTC-3′, R: 5′-GCTCATACGGTCAGCGATAC-3′) expression was used as an internal control.

### Phenotypic analysis under salt-alkaline stress

For the root length assay, the 11-day-old WT and OX seedlings grown on normal 1/2 MS medium were transferred to fresh medium with either 0, or 100, or 115 mM NaCl or with 0, or 8, or 9 mM NaHCO_3_. After vertical growth for another 7 days, the root length was measured. Fifteen seedlings of each line were used for each experiment and the experiments were repeated for three times.

For adult stage, WT and OX were directly grown in soil pots under control conditions for 4 weeks. Then plants were watered with 150 mM NaHCO_3_ (pH = 9.0) solution for salt-alkaline treatment for additional 15 days. Photographs were taken to record plant growth performance, and the survival rates were recorded.

For measurement of Na^+^ and K^+^ content, Arabidopsis leaves were harvested and dried at 80 °C in the oven, ground into powder, and digested with 1 mol L^−1^ HCl overnight. The mixture was centrifuged at 11000 rpm for 3 min, diluted with sterilized milli-Q water and analyzed for Na^+^ and K^+^ content in flame photometer (Aosong 6400A, Shandong, China) as described.

All of the above numerical data were subjected to statistical analyses using EXCEL 2010 and SPSS 17.0 (SPSS, Chicago, USA) statistical software by one-way ANOVA and Student’s t-test.

## Electronic supplementary material

Supplementary information
