# Supplementary material for: GsCHX19.3, a member of cation/H+ exchanger superfamily from wild soybean contributes to high salinity and carbonate alkaline tolerance
Source: Sci Rep. 2017 Aug 25;7:9423. doi: 10.1038/s41598-017-09772-3 (PMC5573395; doi:10.1038/s41598-017-09772-3)
Supplement: Supplementary file 1 — Supplementary information [file 41598_2017_9772_MOESM1_ESM.doc]

**Title Page**

***GsCHX19.3*, a member of cation/H^+^ exchanger superfamily from wild soybean contributes to high salinity and carbonate alkaline tolerance**

**Bowei** **Jia****^1, 2^, Mingzhe Sun****^1, 2^, Huizi** **DuanMu^1^, Xiaodong Ding^1^, Beidong Liu^3^, Yanming Zhu****^1*^, Xiaoli Sun^2*^**

**^1^*Key Laboratory of Agricultural Biological Functional Genes, Northeast Agricultural University, Harbin, 150030, P.R. China***

**^2^*Crop Stress Molecular Biology Laboratory, Heilongjiang Bayi Agricultural University, Daqing, 163319, P.R. China***

**^3^*Department of Chemistry and Molecular Biology, University of Gothenburg, Box 462, Medicinaregatan 9ES-413 90, Gothenburg, Sweden***

**Table S1. List of 55 *GsCPAs* and their sequence details.**

| **No.** | **Gene name** | **Accession** | **Sequence length** | | | **Molecular weight (Da)** | **Isoelectric point** | **TMs^a^** | **Predicted location^b^** | **Na^+^/H^+^ exchanger^c^** |
| --- | --- | --- | --- | --- | --- | --- | --- | --- | --- | --- |
|  |  |  | **DNA**  **（bp）** | **cDNA**  **（bp）** | **Protein（aa）** |  |  |  |  |  |
| **1** | ***GsCHX3*** | **KHN19250.1** | **2924** | **2103** | **700** | **78315.31** | **6.50** | **9** | **ERM, PM** | **1-363** |
| **2** | ***GsCHX4.1*** | **KHN35660.1** | **4063** | **2334** | **777** | **86848.86** | **6.35** | **11** | **PM, CTM** | **32-430** |
| **3** | ***GsCHX4.2*** | **KHN25997.1** | **2515** | **1578** | **525** | **57739.95** | **9.35** | **13** | **PM, CTM** | **70-474** |
| **4** | ***GsCHX4.3*** | **KHN25998.1** | **3314** | **2439** | **812** | **89398.32** | **6.40** | **11** | **PM, CTM** | **69-473** |
| **5** | ***GsCHX4.4*** | **KHN35446.1** | **5027** | **2385** | **794** | **88325.10** | **6.17** | **11** | **CTM, PM** | **60-453** |
| **6** | ***GsCHX14.1*** | **KHN22784.1** | **2605** | **2148** | **715** | **80426.43** | **6.87** | **10** | **PM** | **13-376** |
| **7** | ***GsCHX14.2*** | **KHN02117.1** | **2703** | **246** | **819** | **91544.24** | **9.26** | **12** | **PM, CTM** | **73-463** |
| **8** | ***GsCHX14.3*** | **KHN19136.1** | **3659** | **2457** | **818** | **92068.12** | **8.23** | **10** | **CTM, PM** | **47-442** |
| **9** | ***GsCHX14.4*** | **KHN19137.1** | **3333** | **2454** | **817** | **91402.07** | **6.86** | **11** | **CTM, PM, MIM** | **50-442** |
| **10** | ***GsCHX14.5*** | **KHN04798.1** | **3197** | **2301** | **766** | **85896.33** | **6.19** | **10** | **CTM, PM, MIM** | **3-390** |
| **11** | ***GsCHX14.6*** | **KHN31319.1** | **3116** | **2316** | **771** | **85754.78** | **5.79** | **10** | **CTM, PM** | **1-386** |
| **12** | ***GsCHX15.1*** | **KHN31156.1** | **3406** | **2454** | **817** | **89054.77** | **5.21** | **10** | **PM, CTM** | **41-434** |
| **13** | ***GsCHX15.2*** | **KHN09617.1** | **2421** | **2133** | **710** | **76801.92** | **5.05** | **10** | **PM, CTM** | **9-379** |
| **14** | ***GsCHX15.3*** | **KHN34084.1** | **2605** | **2223** | **740** | **81008.88** | **5.84** | **11** | **ERM, PM** | **1-337** |
| **15** | ***GsCHX15.4*** | **KHN33638.1** | **2307** | **2220** | **739** | **81792.48** | **6.32** | **10** | **PM, ERM** | **1-336** |
| **16** | ***GsCHX18.1*** | **KHN25663.1** | **3185** | **2421** | **806** | **87160.87** | **8.25** | **12** | **CTM, PM** | **38-431** |
| **17** | ***GsCHX18.2*** | **KHN35057.1** | **3155** | **2376** | **791** | **85835.49** | **8.64** | **12** | **PM, CTM** | **24-417** |
| **18** | ***GsCHX19.1*** | **KHN33866.1** | **2825** | **2256** | **751** | **81731.10** | **8.64** | **9** | **PM** | **2-364** |
| **19** | ***GsCHX19.2*** | **KHN45029.1** | **3509** | **2442** | **813** | **88390.13** | **9.00** | **11** | **PM** | **38-427** |
| **20** | ***GsCHX19.3*** | **KHN17780.1** | **3649** | **2403** | **800** | **87095.48** | **8.85** | **11** | **PM** | **25-415** |
| **21** | ***GsCHX20*** | **KHN09423.1** | **7704** | **2436** | **811** | **89430.59** | **8.58** | **10** | **PM** | **30-428** |
| **22** | ***GsCHX23.1*** | **KHN46763.1** | **3626** | **2493** | **830** | **92878.75** | **8.69** | **10** | **CTM, PM** | **51-444** |
| **23** | ***GsCHX23.2*** | **KHN03956.1** | **2653** | **2445** | **814** | **89912.86** | **5.74** | **12** | **CTM, PM** | **35-429** |
| **24** | ***GsCHX23.3*** | **KHN43324.1** | **2628** | **2445** | **814** | **90020.20** | **5.82** | **12** | **CTM, PM** | **35-429** |
| **25** | ***GsCHX23.4*** | **KHN00118.1** | **2624** | **2448** | **815** | **90075.08** | **5.87** | **12** | **CTM, PM** | **34-430** |
| **26** | ***GsCHX23.5*** | **KHN41525.1** | **3652** | **2484** | **827** | **91389.83** | **6.35** | **10** | **PM, CTM** | **53-447** |
| **27** | ***GsCHX23.6*** | **KHN34724.1** | **3268** | **2511** | **836** | **92462.59** | **5.53** | **12** | **PM** | **43-435** |
| **28** | ***GsCHX24.1*** | **KHN22792.1** | **2469** | **2097** | **698** | **78369.70** | **6.16** | **8** | **PM** | **1-361** |
| **29** | ***GsCHX24.2*** | **KHN02110.1** | **2422** | **1902** | **633** | **70599.96** | **5.93** | **12** | **ERM, PM** | **104-297** |
| **30** | ***GsCHX26.1*** | **KHN31313.1** | **3316** | **2421** | **806** | **89828.41** | **6.27** | **10** | **CTM, PM** | **48-436** |
| **31** | ***GsCHX26.2*** | **KHN31312.1** | **4646** | **2628** | **875** | **97385.26** | **7.27** | **9** | **PM, CTM** | **124-486** |
| **32** | ***GsCHX28.1*** | **KHM99196.1** | **3530** | **2103** | **700** | **77659.89** | **6.15** | **7** | **PM** | **2-335** |
| **33** | ***GsCHX28.2*** | **KHN11691.1** | **2337** | **2214** | **737** | **80436.22** | **6.71** | **11** | **PM** | **1-377** |
| **34** | ***GsCHX28.3*** | **KHN32415.1** | **2920** | **2208** | **735** | **80979.75** | **6.23** | **10** | **PM** | **5-348** |
| **35** | ***GsKEA2.1*** | **KHM99400.1** | **12895** | **3330** | **1109** | **118856.02** | **4.83** | **12** | **PM, CTM** | **508-883** |
| **36** | ***GsKEA2.2*** | **KHN29378.1** | **12444** | **3519** | **1172** | **126502.54** | **5.09** | **10** | **PM, CTM** | **601-842** |
| **37** | ***GsKEA2.3*** | **KHN04650.1** | **9640** | **3138** | **1045** | **112438.91** | **4.86** | **12** | **PM, CTM** | **444-819** |
| **38** | ***GsKEA2.4*** | **KHN27308.1** | **15064** | **4050** | **1349** | **146500.19** | **5.01** | **12** | **PM, CTM** | **748-1123** |
| **39** | ***GsKEA3*** | **KHN46227.1** | **9337** | **2424** | **807** | **87751.94** | **5.77** | **10** | **PM** | **117-504** |
| **40** | ***GsKEA4*** | **KHM99845.1** | **7643** | **1569** | **522** | **56117.12** | **5.83** | **12** | **PM, CTM** | **104-474** |
| **41** | ***GsKEA5.1*** | **KHN24404.1** | **11470** | **1821** | **606** | **66418.28** | **6.76** | **14** | **PM** | **170-540** |
| **42** | ***GsKEA5.2*** | **KHN27840.1** | **8997** | **1497** | **498** | **53783.66** | **6.34** | **12** | **PM, CTM** | **92-462** |
| **43** | ***GsKEA6.1*** | **KHN37024.1** | **10713** | **1785** | **594** | **64277.43** | **6.07** | **13** | **PM** | **175-545** |
| **44** | ***GsKEA6.2*** | **KHN17221.1** | **10180** | **1560** | **519** | **56194.14** | **5.95** | **12** | **PM, CTM** | **100-470** |
| **45** | ***GsNHX2.1*** | **KHN20184.1** | **4762** | **1641** | **546** | **60102.35** | **6.78** | **10** | **CTM, PM** | **24-444** |
| **46** | ***GsNHX2.2*** | **KHN16165.1** | **5172** | **1521** | **506** | **55579.87** | **6.17** | **9** | **CTM, PM** | **99-404** |
| **50** | ***GsNHX2.3*** | **KHN43237.1** | **3549** | **1644** | **547** | **60740.20** | **8.99** | **9** | **PM, CTM** | **23-444** |
| **47** | ***GsNHX2.4*** | **KHN04040.1** | **3832** | **1644** | **547** | **60791.19** | **8.77** | **8** | **PM, CTM** | **23-444** |
| **48** | ***GsNHX2.5*** | **KHN03394.1** | **3696** | **1518** | **505** | **56068.35** | **8.66** | **8** | **CTM, PM** | **25-418** |
| **52** | ***GsNHX2.6*** | **KHN03007.1** | **3748** | **1584** | **527** | **58829.46** | **9.02** | **9** | **CTM, PM** | **31-438** |
| **53** | ***GsNHX4*** | **KHM99503.1** | **5392** | **1590** | **529** | **58904.42** | **7.69** | **10** | **PM** | **24-447** |
| **49** | ***GsNHX6.1*** | **KHN40217.1** | **5839** | **1338** | **445** | **49098.98** | **5.76** | **9** | **ERM, PM** | **20-370** |
| **51** | ***GsNHX6.2*** | **KHN14386.1** | **6525** | **1563** | **520** | **56967.79** | **5.44** | **10** | **PM, CTM** | **29-423** |
| **54** | ***GsNHX6.3*** | **KHN40748.1** | **7018** | **1380** | **459** | **50186.77** | **5.08** | **8** | **PM** | **44-365** |
| **55** | ***GsNHX7*** | **KHN28893.1** | **12423** | **3426** | **1141** | **126206.35** | **6.30** | **12** | **PM** | **36-448** |

PM: plasma membrane CTM: chloroplast thylakoid membrane ERM: endoplasmic reticulum membrane MIM: mitochondrial inner membrane

^a^ TMs, number of transmembrane domain.

^b^ Localization of GsCPA proteins were predicted by PSORT (http://psort.hgc.jp/).

^c^ Na^+^/H^+^ exchange domain was supported by PFAM (http://pfam.xfam.org/).

**Table S2. The raw expression data of *GsCHXs* and *GsGAPDH***.

| **No.** | **Gene Name** | **Expression value (RPKM)** | | | | | |
| --- | --- | --- | --- | --- | --- | --- | --- |
|  |  | **0 h** | **1 h** | **3 h** | **6 h** | **12 h** | **24 h** |
| **1** | ***GsCHX28.1*** | **0** | **0.42347** | **0.38951** | **0.18207** | **0** | **0.09892** |
| **2** | ***GsCHX28.2*** | **0** | **0.21723** | **0** | **0** | **0** | **0.10149** |
| **3** | ***GsCHX28.3*** | **0** | **0.66485** | **0** | **0** | **0** | **0** |
| **4** | ***GsCHX19.1*** | **0** | **0** | **0** | **0** | **0** | **0** |
| **5** | ***GsCHX19.2*** | **0.72** | **0.43797** | **2.31749** | **0.5649** | **0.91764** | **0.51155** |
| **6** | ***GsCHX19.3*** | **1.98448** | **9.02605** | **24.9067** | **7.18351** | **2.4143** | **1.0767** |
| **7** | ***GsCHX18.1*** | **6.26775** | **13.4292** | **14.3976** | **16.8434** | **13.2113** | **12.1488** |
| **8** | ***GsCHX18.2*** | **5.70653** | **24.0167** | **10.4866** | **10.2054** | **7.72026** | **6.20012** |
| **9** | ***GsCHX20.1*** | **0.72913** | **0.60368** | **3.25228** | **1.70564** | **0.7228** | **0.24175** |
| **10** | ***GsCHX15.1*** | **0** | **0.10524** | **0.38719** | **0.18099** | **0.378** | **0** |
| **11** | ***GsCHX15.2*** | **0** | **0.10705** | **0** | **0** | **0** | **0** |
| **12** | ***GsCHX15.3*** | **0** | **0** | **0** | **0** | **0** | **0** |
| **13** | ***GsCHX15.4*** | **0** | **0.09736** | **0** | **0** | **0** | **0** |
| **14** | ***GsCHX23.6*** | **0** | **0** | **0** | **0** | **0** | **0** |
| **15** | ***GsCHX23.5*** | **0** | **0.11439** | **0** | **0** | **0** | **0** |
| **16** | ***GsCHX23.4*** | **0** | **0** | **0.09962** | **0** | **0** | **0** |
| **17** | ***GsCHX23.3*** | **0** | **0.21774** | **0** | **0** | **0** | **0** |
| **18** | ***GsCHX23.2*** | **0.30682** | **0.21774** | **0** | **0** | **0** | **0.10173** |
| **19** | ***GsCHX23.1*** | **0** | **0** | **0** | **0** | **0** | **0** |
| **20** | ***GsCHX24.1*** | **0** | **0** | **0** | **0** | **0.12907** | **0** |
| **21** | ***GsCHX24.2*** | **0** | **0** | **0** | **0** | **0** | **0** |
| **22** | ***GsCHX26.1*** | **0** | **0** | **0** | **0** | **0** | **0** |
| **23** | ***GsCHX26.2*** | **0** | **0** | **0** | **0** | **0** | **0** |
| **24** | ***GsCHX14.6*** | **0** | **0.23329** | **0** | **0.1003** | **0** | **0** |
| **25** | ***GsCHX14.5*** | **0** | **0** | **0.21137** | **0.1976** | **0** | **0** |
| **26** | ***GsCHX14.4*** | **0** | **0** | **0** | **0** | **0** | **0** |
| **27** | ***GsCHX14.3*** | **0.22117** | **0.11772** | **0.32484** | **0** | **0.28188** | **0.22** |
| **28** | ***GsCHX14.2*** | **0** | **0.10572** | **0** | **0** | **0** | **0** |
| **29** | ***GsCHX14.1*** | **0** | **0** | **0** | **0** | **0** | **0** |
| **30** | ***GsCHX3*** | **0** | **0** | **0** | **0** | **0** | **0** |
| **31** | ***GsCHX4.4*** | **0** | **0** | **0** | **0** | **0** | **0** |
| **32** | ***GsCHX4.3*** | **0** | **0** | **0** | **0** | **0** | **0** |
| **33** | ***GsCHX4.2*** | **0** | **0** | **0** | **0** | **0** | **0** |
| **34** | ***GsCHX4.1*** | **0** | **0.12541** | **0** | **0** | **0** | **0** |
| **35** | ***GsGAPDH*** | **326.578** | **247.91** | **247.902** | **306.869** | **232.21** | **294.03** |

RPKM: reads per kilo bases per million reads

**Figure S1**


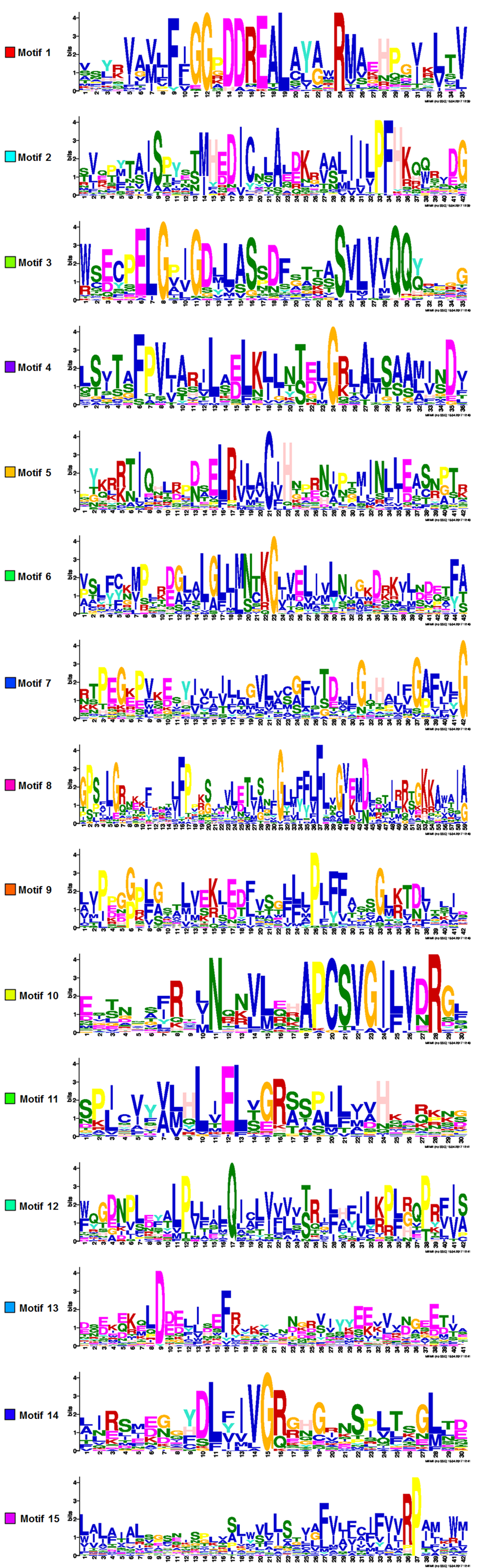


**Figure S1.** **Details of 15 conserved domains across CHX proteins in soybean.** The sequence logos were based on alignments of 34 GsCHXs. The bit score indicated the information content for each position in the sequence.

**Figure S2**


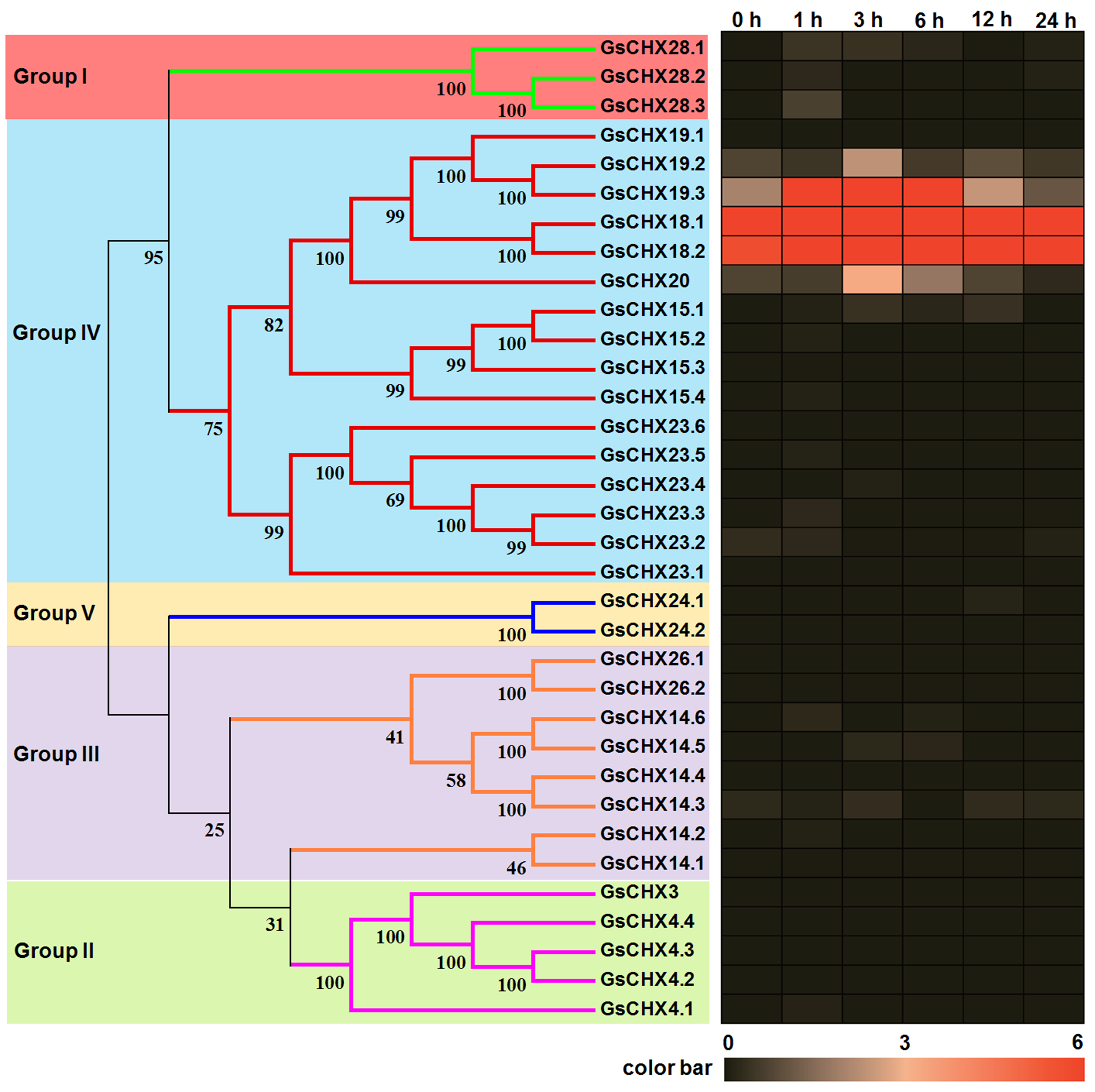


**Figure S2. Expression profiles of *GsCHXs* under carbonate alkaline stress.** A heatmap showing expression profiles of *GsCHXs* in response to carbonate alkaline treatment was created according to our previous RNA-seq data.

**Figure S****3**


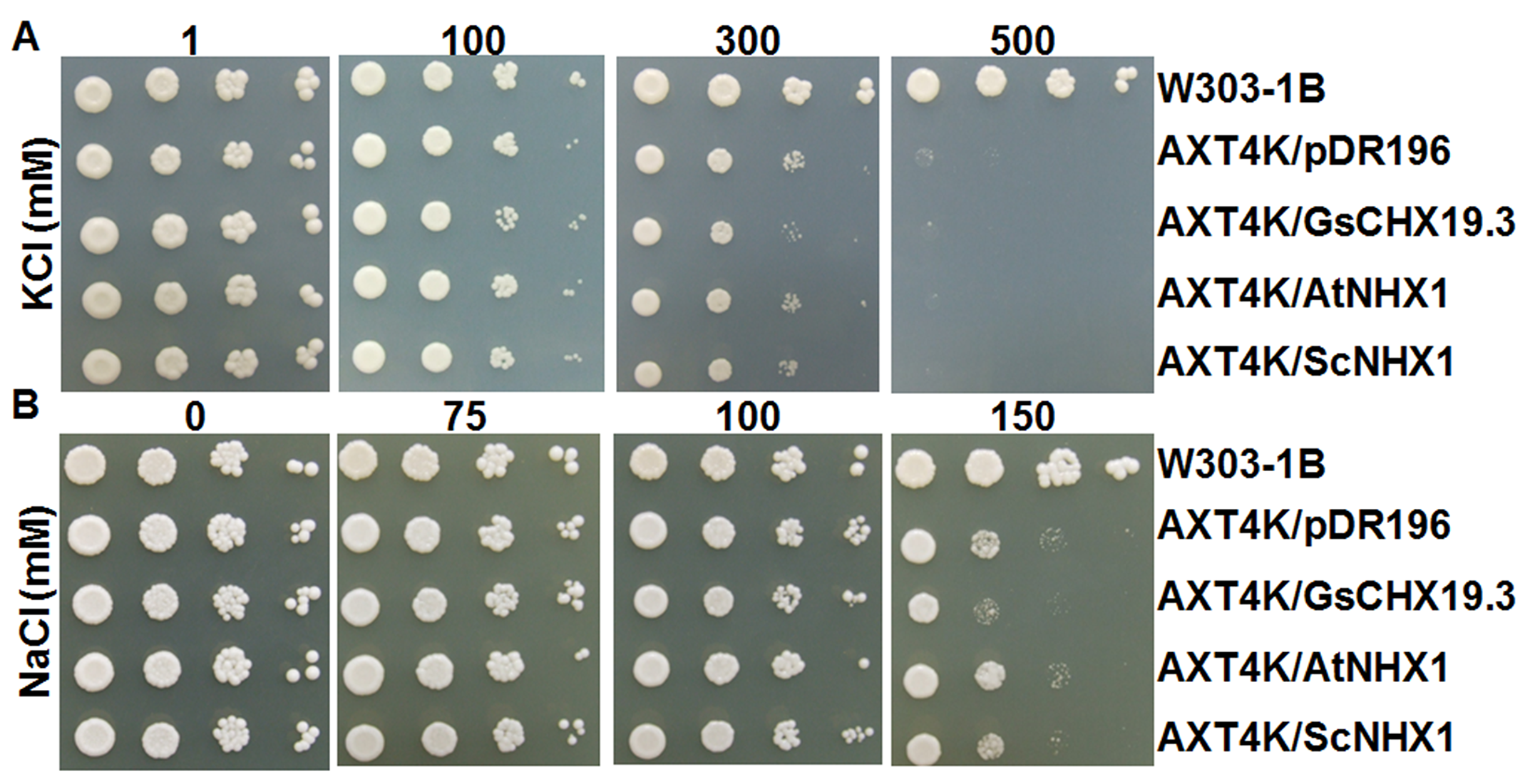


**Figure S3.** ***GsCHX19.3* couldn’t confer resistance to high salt stress in yeast mutant AXT4K.** Transformed strains were diluted and spotted onto AP plates with different concentrations of KCl (1, 100, 300 or 500 mM) (A), or YPDA plates containing different concentrations of NaCl (0, 75, 100 or 150 mM) (B). All the strains were grown at 30 ℃ for 3 days.
